# Supplementary material for: Inflammatory and Redox Responses During Medical Treatment of Open-Cervix Pyometra in Female Dogs: A Prospective Study
Source: Animals (Basel). 2025 Dec 8;15(24):3531. doi: 10.3390/ani15243531 (PMC12729436; doi:10.3390/ani15243531)
Supplement: Supplementary file 1 [file animals-15-03531-s001.zip › animals-3988889-supplementary.pdf]

Table S1. Case population (breed, age and weight) and clinical signs at the time of pyometra diagnosis in bitches of the Aglepristone and Aglepristone + Prostaglandin groups.

Group	Breed	Age (yo)	Weight (kg)	Clinical Signs
**Aglepristone Group**	Dachshund	8	8.8	purulent vaginal discharge, lethargy, inappetence and dehydration
	Weimaraner	8	44	purulent vaginal discharge, lethargy, inappetence and dehydration
	Lhasa Apso	9	3.6	purulent vaginal discharge, lethargy, inappetence and dehydra622tion
	Mixed-breed	7	11.3	purulent vaginal discharge, lethargy, inappetence, depression and dehydration
	Mixed-breed	8	22.3	purulent vaginal discharge, lethargy, inappetence and dehydration
**Aglepristone + Prostaglandin Group**	Boxer	9	23	purulent vaginal discharge, lethargy, inappetence and dehydration
	Mixed-breed	10	16.4	purulent vaginal discharge, lethargy, inappetence and dehydration
	Mixed-breed	10	13.3	purulent vaginal discharge, lethargy, inappetence and dehydration
	Mixed-breed	4	11.4	purulent vaginal discharge, lethargy, inappetence and dehydration
	Labrador	9	35	purulent vaginal discharge, lethargy, inappetence and dehydration

Table S2. Kidney function test at the time of pyometra diagnosis in bitches of the Aglepristone and Aglepristone + Prostaglandin groups.

Group	Creatinine (0.5–1.5 mg/dL)	BUN (21 – 59.9 mg/dL)
**Aglepristone Group**	0.5	20
	1	7
	0.5	20
	**2.22**	**73.6**
	0.83	30.8
**Aglepristone + Prostaglandin Group**	1.52	*
	0.6	17
	0.89	15
	0.8	15
	1.2	25

BUN - Blood urea nitrogen
Bold values represent results out of the reference range for dogs.
* Data not available.

Table S3. Complete Blood Count at the time of pyometra diagnosis in bitches of the Aglepristone and Aglepristone + Prostaglandin groups.

Group	WBC (6-15x10^3/µL)	Neutrophils (3-11 x10^3/µL)	Eosinophils (0-1300 /µL)	Lymphocytes (1500 - 5000/µL)	Monocyte count (0-800/µL)	Platelets (200-600x10^3/µL)	RBC (5-8x10^6/µL)	Hematocrit (37–57%)	Hemoglobin (12-18g/dL)	MCV (60-77fl)	MCH (22-27pg)	MCHC (31-36%)
**Aglepristone Group**	**25200**	**16632**	1008	**6552**	**1008**	246	**8.81**	53	**18.8**	60	**21.3**	**35.5**
	**25430**	**20620**	240	**1270**	**3270**	**188**	6.02	43.3	**10.5**	72	24.6	34.1
	**23400**	**19188**	234	2340	**936**	**181**	**4.86**	**35**	**11.5**	72	23.7	32.9
	**62200**	**51626**	622	**6220**	**1866**	**90**	5.5	38	13.6	69.1	24.7	**35.8**
	**36100**	**31768**	722	2166	**1444**	**22.5**	7.3	47	15.5	65	**21**	33
**Aglepristone + Prostaglandin Group**	**48600**	**38880**	972	3402	**5346**	339	5.32	37	**11.5**	69.5	**21.6**	31.1
	**25900**	**22792**	259	1813	777	264	**4.66**	**30**	**10**	64	**21.5**	33.3
	**54400**	**33184**	1088	2720	**2720**	302	**4.24**	**31.2**	**10.4**	73.8	24.5	33.3
	**43000**	**34830**	0	**6880**	**1290**	**198**	**4.49**	**30**	**10.4**	67	23.2	34.7
	**35400**	**30090**	0	2478	**2832**	204	6.33	44	15	69.5	23.7	34.1

WBC - White blood cell count, RBC – red blood cell count; MCV - mean corpuscular volume, MCH - mean corpuscular hemoglobin, MCHC - mean corpuscular hemoglobin concentration.
Bold values represent results out of the reference range for dogs
